# Subcellular Sorting of the G-Protein Coupled Mouse Somatostatin Receptor 5 by a Network of PDZ-Domain Containing Proteins

**DOI:** 10.1371/journal.pone.0088529

**Published:** 2014-02-11

**Authors:** Carola Bauch, Judith Koliwer, Friedrich Buck, Hans-Hinrich Hönck, Hans-Jürgen Kreienkamp

**Affiliations:** 1 Institut für Humangenetik, Universitätsklinikum Hamburg-Eppendorf, Hamburg, Germany; 2 Institut für klinische Chemie, Universitätsklinikum Hamburg-Eppendorf, Hamburg, Germany; German Institute for Human Nutrition, Germany

## Abstract

PSD-95/discs large/ZO-1 (PDZ) domain proteins integrate many G-protein coupled receptors (GPCRs) into membrane associated signalling complexes. Additional PDZ proteins are involved in intracellular receptor trafficking. We show that three PDZ proteins (SNX27, PIST and NHERF1/3) regulate the mouse somatostatin receptor subtype 5 (SSTR5). Whereas the PDZ ligand motif of SSTR5 is not necessary for plasma membrane targeting or internalization, it protects the SSTR5 from postendocytic degradation. Under conditions of lysosomal inhibition, recycling of the SSTR5 to the plasma membrane does not depend on the PDZ ligand. However, recycling of the wild type receptor carrying the PDZ binding motif depends on SNX27 which interacts and colocalizes with the receptor in endosomal compartments. PIST, implicated in lysosomal targeting of some membrane proteins, does not lead to degradation of the SSTR5. Instead, overexpressed PIST retains the SSTR5 at the Golgi. NHERF family members release SSTR5 from retention by PIST, allowing for plasma membrane insertion. Our data suggest that PDZ proteins act sequentially on the GPCR at different stages of its subcellular trafficking.

## Introduction

Signalling by G-protein coupled receptors (GPCRs) is controlled by a variety of cellular mechanisms. Specific protein interactions determine the incorporation of receptors into signalling complexes [1], [2], lead to desensitization of receptors after agonist exposure, and initiate receptor internalization, lysosomal degradation or recycling [3], [4]. A particular interaction motif which is involved in regulating a number of different receptors is the PSD-95/ZO-1/discs large domain (PDZ domain). PDZ domain containing proteins are typically soluble, cytoplasmic proteins which associate with C-terminal PDZ-ligand motifs of membrane proteins [5]. Due to the promiscuity of PDZ domain mediated interactions, multiple proteins may interact with any given receptor carrying a PDZ ligand [6]. Thus it is difficult to determine which PDZ protein is functionally relevant for individual receptors. Some PDZ domain proteins are attached to intracellular organelles, including the Golgi-associated GRASP and PIST/GOPC [7], [8], as well as the endosomal sorting nexin 27 (SNX27) [4], [9], [10]. Other proteins containing multiple interaction motifs such as PSD-95, MUPP1 or NHERF family members act as scaffolds by integrating GPCRs into larger protein complexes at the plasma membrane [2], [11], [12], [13]. So far it is unclear whether these receptor/scaffold protein complexes are formed spontaneously at the plasma membrane, or whether they are preformed early in the biosynthetic pathway of the receptor, and then transported to the plasma membrane.

Somatostatin receptor subtype 5 (SSTR5) is an inhibitory G-protein coupled receptor which regulates hormone secretion in the pituitary and in pancreatic islets [14]. We have recently shown that it interacts with the PDZ domains of the Golgi-associated protein PIST (also termed GOPC, CAL or FIG) as well as the scaffold protein NHERF3/PDZ-K1 via its C-terminal PDZ ligand motif [8], [15]. PIST associates with a number of GPCRs as well as other membrane proteins such as the cystic fibrosis transmembrane regulator (CFTR). Overexpressed PIST, due to its anchorage at the Golgi, may retain GPCRs in the Golgi apparatus [8], [16]. The functional relevance of this observation remained is unclear. In parallel, several publications have shown that PIST may direct an associated membrane protein (namely the CFTR) towards lysosomal degradation [17], [18], [19]. Work on the CFTR also suggested that escape of the CFTR from degradation by PIST is possible only due to the low affinity of the CFTR PDZ ligand for the PIST PDZ domain [19]. In contrast to the CFTR, SSTR5 has a rather high affinity for PIST, suggesting that SSTR5 might be even more susceptible to the degradative function of PIST. On the other hand, our previous work suggested a role for the PDZ ligand in receptor recycling after agonist-induced endocytosis [8].

Here we identify additional interaction partners of the PDZ ligand motif of the SSTR5 and attempt a thorough analysis of the role of PDZ type interactions for the stability, subcellular localization, endocytic trafficking and incorporation into signalling complexes of the SSTR5. We find that the PDZ ligand motif stabilizes, rather than destabilizes the SSTR5. In addition we show that at least three different types of PDZ domain proteins cooperate in determining the subcellular localization and plasma membrane availability of the SSTR5.

## Results

### Role of the PDZ ligand motif in receptor turnover

**Figure 1:**
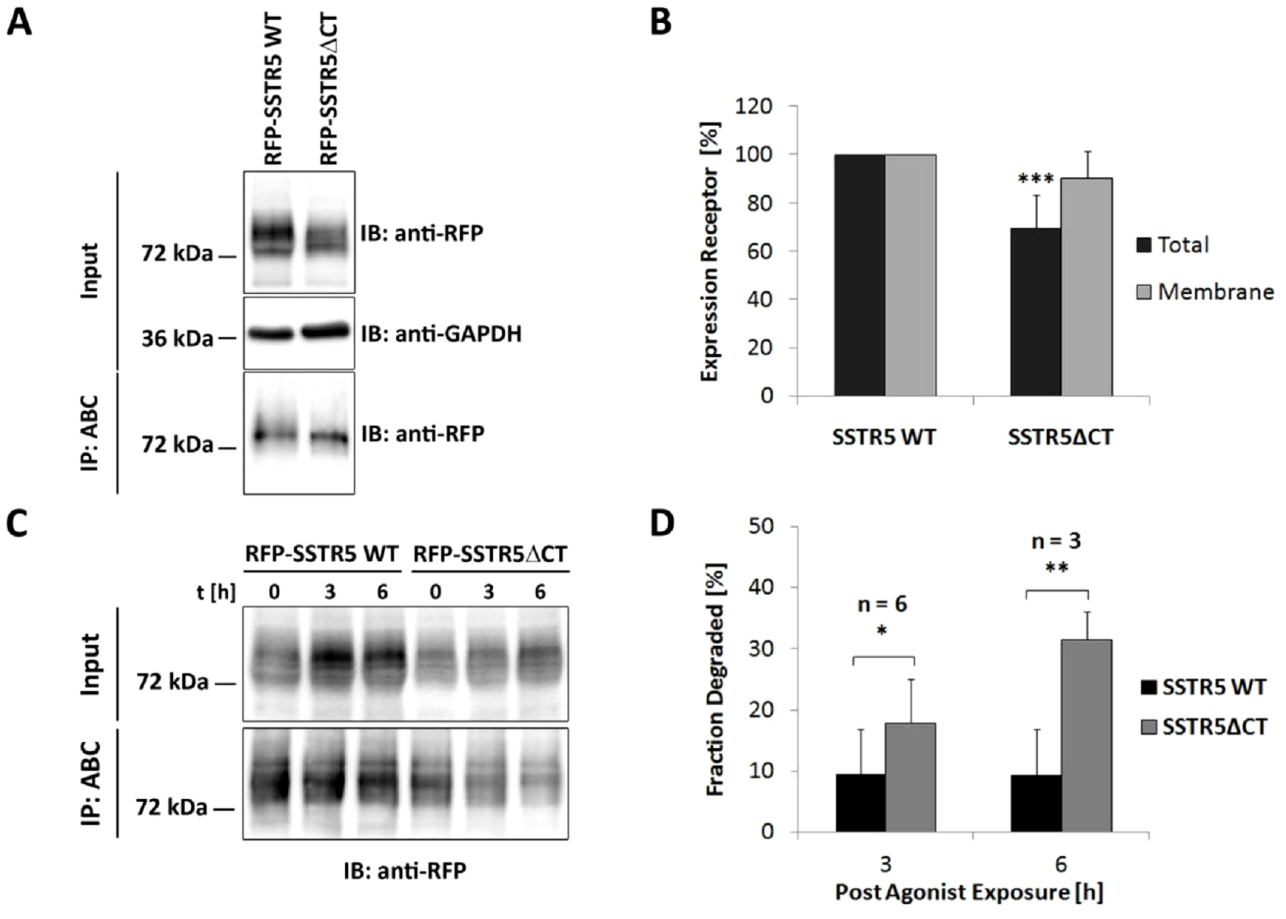
The PDZ ligand motif of the SSTR5 protects against receptor degradation. A. HEK293 Flp-In cells expressing mRFP fused to wt SSTR5, or to a C-terminally truncated SSTR5 variant lacking the PDZ ligand, were subjected to cell surface biotinylation, followed by lysis and purification of biotinylated proteins using streptavidin agarose (ABC: avidin biotin complex). Input and precipitate samples were analyzed by western blotting using anti-mRFP, as well as anti-GAPDH for input samples. B. Quantification of the data shown in A; total SSTR5 levels were determined by normalizing receptor signals on the intensity of GAPDH signals (***, significantly different from wild type; p<0.001; n = 13;). Cell surface levels were calculated by normalizing the signal of ABC-purified receptors against the total cellular receptor signal (n = 8). Data are represented in relation to those obtained with wild type (100%). C. Receptor expressing cells were subjected to cell surface biotinylation; after treatment with SST28 for 15 min, cells were treated with glutathione to strip biotin from remaining cell surface receptors. After further incubation at 37°C for the times indicated, biotinylated receptors were purified (ABC) and quantified by Western Blotting using anti-mRFP. D. Quantification of the data shown in C. Data are presented as the percentage of internalized receptors (100% at time  = 0) lost during the incubation period (*, **, different from wt, p<0.05 and p<0.01, respectively).

We analyzed whether the PDZ ligand motif of the SSTR5 affects the stability of the receptor, assuming that an eventual targeting of the receptor to the lysosome by PIST would be affected by loss of the PDZ binding motif at the C-terminus of the receptor. Therefore we generated two cell lines, one expressing the wild type receptor and one expressing a receptor lacking the last 4 amino acids (i.e. the PDZ ligand). We used FlpIn-HEK293 cells to make sure that the expression cassette is in each case inserted in the same gene locus, thereby ensuring similar expression levels. Receptor constructs carried a signal peptide, followed by the mRFP sequence in frame with the SSTR5 cDNA, thus allowing for easy detection of receptors. When whole cell lysates from both lines were analyzed by Western blotting, we observed that the truncated receptor (SSTR5ΔCT) was produced at significantly lower levels (70% of the wild type; Fig. 1A,B). On the other hand, cell surface biotinylation experiments showed that both receptor variants are efficiently transported to the cell surface (Fig. 1A,B).

To determine whether both receptor variants are differentially targeted towards lysosomal degradation after agonist dependent endocytosis, cells were subjected to surface biotinylation, followed by treatment with the agonist SST-28 for 15 min. Biotin was removed from receptors which were still at the cell surface at this time point by stripping with glutathione, followed by further incubation at 37°C for 3 or 6 hours. After cell lysis, biotinylated proteins were purified using streptavidin agarose, followed by Western Blot analysis using anti-mRFP detection. By this procedure we ensured that only the fate of those receptors was analyzed which had undergone endocytosis during agonist treatment. Here we observed that the truncated receptor was degraded over time, with more than 30% of the receptor lost after 6 hours. In contrast, the wt receptor remained stable during this time period (Fig. 1C,D). Thus, loss of the PDZ ligand motif destabilizes the receptor, allowing for faster degradation after endocytosis.

**Figure 2:**
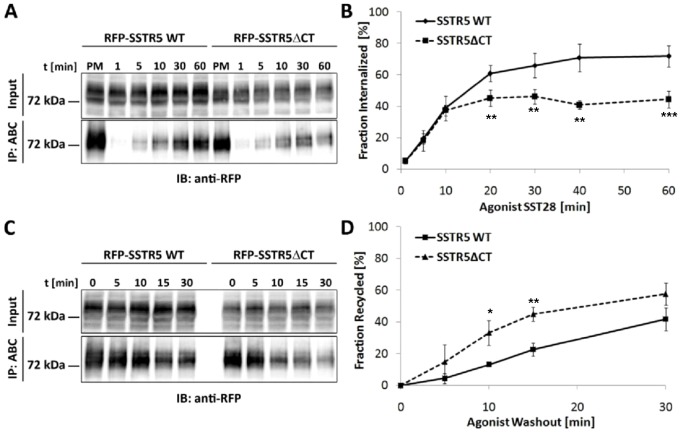
The PDZ ligand motif affects receptor endocytosis and recycling. A. Receptor expressing cells were surface biotinylated; after treatment with SST-28 for the times indicated, cells were treated with glutathione to remove biotin from remaining cell surface receptors. Internalized, biotinylated receptors were purified (ABC) and quantified by Western blotting as before. PM, plasma membrane sample, no agonist treatment, no stripping. B. Quantification of the data shown in A. The signal obtained in the PM control sample was set as the 100% value for each receptor variant. **, ***: significantly different from wt at this time point; p<0.01, p<0.001, respectively. Data are derived from three to five independent experiments for each time point. C. Recycling was analyzed by surface biotinylation of receptor expressing cells, followed by internalization in the presence of agonist (15 min, 1 µM SST28) and stripping with glutathione. Recycled receptors were then stripped again at the time points indicated. To avoid lysosomal degradation as observed in Fig.1, cells were treated with the lysosomal inhibitor leupeptin (100 µM; starting 24 h before the experiment). Intracellular, biotinylated receptors were then purified and analyzed by Western blotting as before. D. Quantitation of the data obtained in C shows that the SSTR5ΔCT receptor recycles faster than the wt receptor. *, **: significantly different from wt at this time point; p<0.05, p<0.01, respectively (t-test); n = 3 for each time point.

In the next steps we analyzed whether the PDZ motif affects either the endocytosis process itself, or postendocytic recycling. Previous work had indicated that lack of the PDZ motif impaired recycling of the receptor to the cell membrane [8], which could be due to defects in recycling or the enhanced lysosomal degradation which we have observed here. To distinguish between these possibilities, experiments were carried out in the presence of the lysosomal inhibitor, leupeptin. Cell surface receptors were biotinylated; cells were treated with agonist for various periods of time, and biotin was removed from remaining cell surface receptors by stripping with glutathione. The remaining biotinylated receptors (representing the internalized pool) were purified by streptavidin agarose and quantified by Western Blot. Here we observed that both receptor variants were internalized rapidly by SST28 treatment, but the extent of internalization was higher for the wild type receptor (Fig. 2A,B). To assess recycling, we internalized biotinylated receptors by agonist treatment for 15 minutes, followed by stripping with glutathione. Cells were returned to 37°C to allow for recycling, and reappearing cell surface receptors were stripped again at certain time points. After lysis the remaining biotinylated receptors were purified and quantified by Western blotting. The mRFP-SSTR5 signal lost in the second stripping procedure was considered as the fraction of receptors which had been recycled, as these receptors could not have been degraded due to lysosomal inhibition. Here we observed that both receptors were recycled, with the SSTR5ΔCT variant exhibiting faster recovery at the cell surface (Fig. 2C,D).

### Identification of additional PDZ domain proteins interacting with the SSTR5

**Figure 3:**
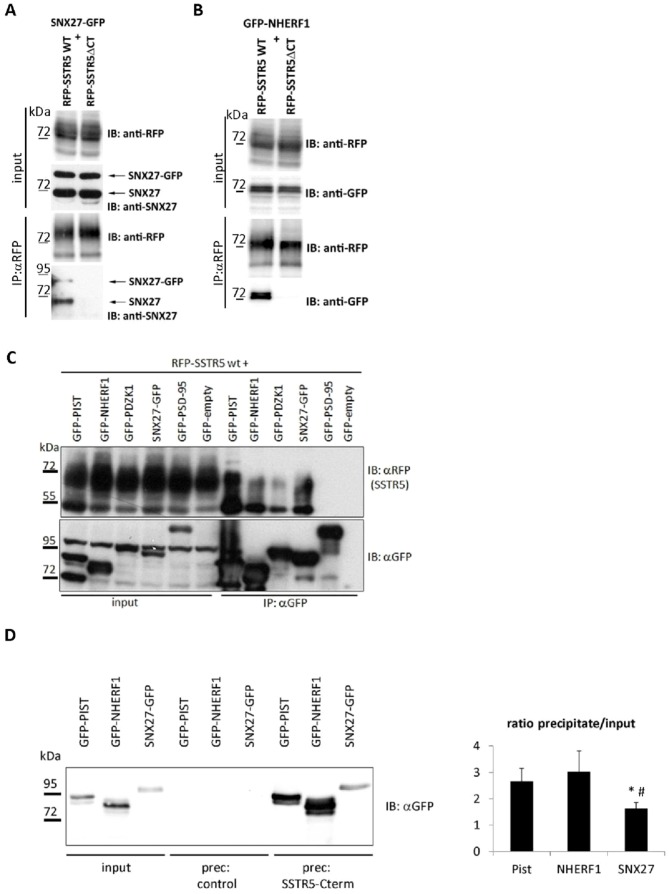
Interaction of the SSTR5 with SNX27 and NHERF1. A. Cells were transfected with expression vectors coding for mRFP-tagged wt SSTR5 or the truncation mutant SSTR5ΔCT, and a GFP-fusion of SNX27. After cell lysis, receptors were precipitated using RFP-trap matrix. Input and precipitate (IP) samples were analyzed by Western Blot using mRFP and SNX27 specific antibodies. The positions of endogenous and GFP-tagged recombinant SNX27 are indicated. B. A GFP-fusion of NHERF1 was coexpressed with either of the two receptor variants. After cell lysis, receptors were precipitated using RFP-trap matrix. Input and precipitate (IP) samples were analyzed by Western Blot using mRFP and GFP specific antibodies. C. mRFP-tagged SSTR5 was coexpressed with GFP-tagged PDZ proteins; cells lysates were subjected to immunoprecipitation using GFP-trap matrix. Input and precipitate samples were analyzed by Western blotting. For A–C, a typical of three experiments is shown in each case. D. GFP-tagged PDZ domain proteins from cell lysates of transfected 293 cells (input) were precipitated with control NHS-sepharose, or sepharose conjugated with the SSTR5 C-terminal peptide carrying the PDZ ligand sequence. Input and precipitate samples were analyzed by Western blotting using anti-GFP antibody. For quantitation, the ratio of precipitate to input signal intensity was determined. *,#, significantly different from PIST and NHERF1, respectively (p<0.05; Anova (p = 0.015), followed by t-test with Bonferroni correction; n = 4).

Based on the differential effects of the PDZ ligand motif we assumed that additional PDZ proteins might be involved in receptor sorting. Therefore we performed peptide affinity chromatography on HEK293 cell lysates, using a synthetic peptide corresponding to the C-terminal tail of the SSTR5, including the PDZ ligand motif (supporting information; Figure S1 in File S1). We identified sorting nexin 27 (SNX27) as a new interactor which contains one Phox domain and, similar to PIST, a single PDZ domain (Figure S1 in File S1). In addition, we had previously shown that PDZ-K1/NHERF3 binds to the SSTR5 C-terminus [8]. NHERF3 is expressed in HEK293 cells at low levels whereas its homologue, NHERF1, has been shown to be present in this cell line [20]. Coexpression/coimmunoprecipitation assays showed that both SNX27 and NHERF1 strongly interact with the SSTR5, whereas the SSTR5ΔCT mutant does not (Fig. 3A,B). In further experiments, the SSTR5 specifically interacted with PIST, SNX27, NHERF1 and NHERF3 but not with PSD-95 carrying three PDZ domains (Fig. 3C). In peptide pulldown experiments using the immobilized C-terminal PDZ ligand we observed that SNX27 interacts less efficiently when compared with PIST and NHERF1, suggesting that the affinity of the SNX27 PDZ domain is lower than that of PIST or NHERF1 (Fig. 3D).

To analyze whether all interaction partners are present in tissues expressing the SSTR5, we compared expression patterns in brain, pituitary and in two cells lines which are known to express the SSTR5, the pancreatic Min-6 line and the mouse pituitary tumour line AtT-20 [8], [15]. Both PIST and SNX27 were found in all samples tested, whereas NHERF1 was detected in brain and rat pituitary, but at rather low levels in mouse pituitary and the two cell lines (Figure S2 in File S1).

**Figure 4:**
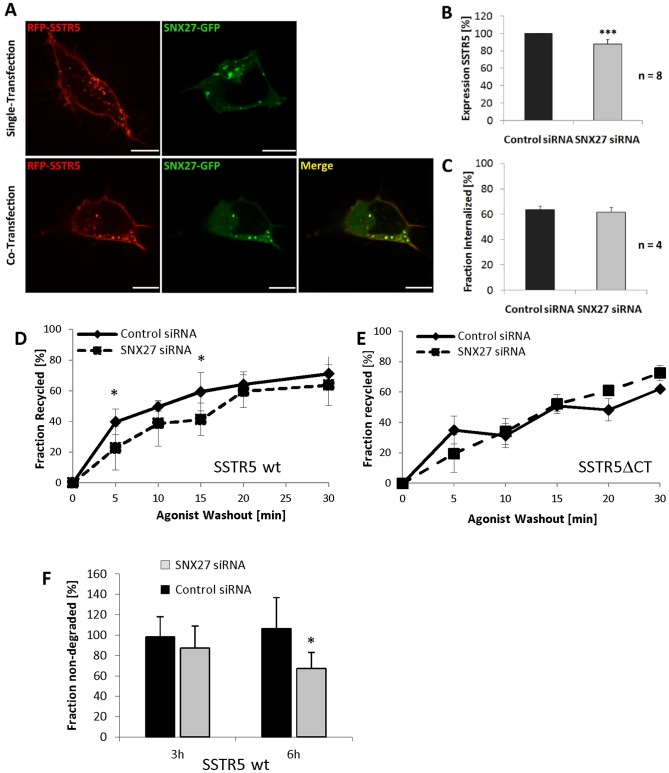
SNX27 is involved in recycling of SSTR5. A. 293 cells were transfected with expression constructs coding for mRFP-tagged wt and mutant SSTR5, and GFP-SNX27. Cells were plated on cover slips and imaged by fluorescence microscopy using mRFP and GFP specific settings. B. mRFP-SSTR5 expressing cells were transfected with SNX27 specific siRNA or control non-specific siRNA; 72 hours after transfection, cells were lyzed and analyzed by Western blotting using mRFP, SNX27 and GAPDH specific antibodies. Reduction of SNX27 levels to 19% of control led to a reduction of SSTR5 levels to 88%. C. SNX27 siRNA treated cells expressing the wt SSTR5 were analyzed for agonist dependent endocytosis as in Fig. 2A and B. The fraction of receptors which have been endocytosed after 15 min of agonist treatment is depicted. No differences in endocytosis efficiency was observed. D. The degradation of endocytosed wt SSTR5 after agonist dependent endocytosis was analyzed by surface biotinylation. Data are represented as the percentage of receptors remaining after the time indicated (*, significantly different, p<0.05, t-test; n = 6). E,F. SNX27 expression was knocked down in cells expressing the wt SSTR5 (E), or the SSTR5ΔCT mutant (F). Recycling of the receptor after agonist-induced internalization was analyzed as described in Fig. 2 by surface biotinylation and stripping of remaining surface receptors with glutathione. Data are presented as the fraction of the internalized receptors which return to the cell surface at a given time point (*, significantly different, p<0.05, Mann-Whitney U-test; n = 4).

Immunocytochemical analysis indicated that SSTR5 and SNX27 colocalize mostly in intracellular vesicular structures which are likely to be endosomes, based on the known localization of SNX27 [10] (Fig. 4A; see also Figure S3 in File S1). To determine the role of SNX27 in SSTR5 trafficking, we established an efficient siRNA based knockdown for the endogenous SNX27 in HEK293 cells (SNX27 protein levels reduced to 19 +/− 3% of control values). This treatment slightly, but significantly reduced the total cellular levels of the receptor (Fig. 4B). SNX27 has been reported to accelerate endocytosis of GIRK/Kir3 channels [9], [10]; however, the rate of agonist dependent endocytosis of the SSTR5 was not affected by loss of SNX27 (Fig. 4C). Instead, knockdown of SNX27 significantly delayed recycling of the SSTR5 after agonist-dependent endocytosis, while recycling of the SSTR5ΔCT mutant was not affected (Fig. 4D,E). In addition, using assays established in Fig. 1, we observed enhanced degradation of endocytosed SSTR5 upon SNX27 knockdown (Fig. 4F). These data are in agreement with previous observations made for the β_2_-adrenergic receptor, [4], [9], suggesting that interaction of SNX27 with the PDZ ligand motif switches trafficking of the receptor from lysosomal targeting to a recycling mode.

### PIST and NHERF1 regulate cell surface availability of SSTR5

**Figure 5:**
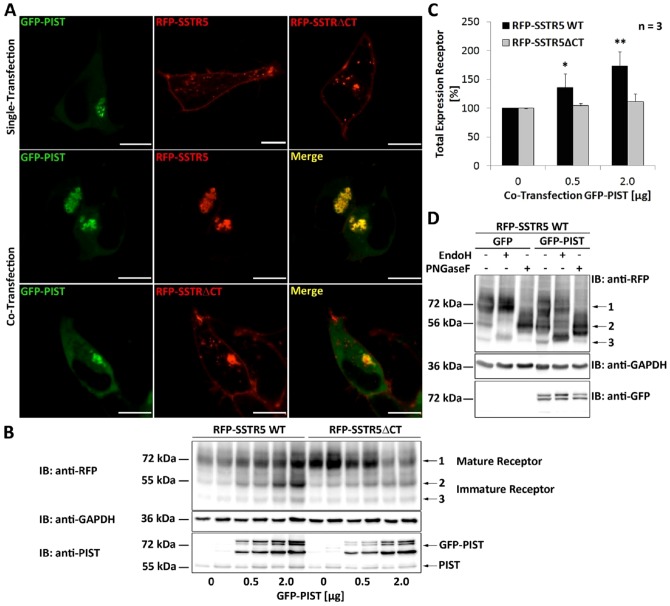
PIST retains the SSTR5 at the TGN. A. HEK cells expressing GFP-PIST and mRFP-SSTR5 either alone or in combination were imaged under a fluorescence microscope. Bar, 10 µm. B. Cells expressing mRFP-SSTR5 or mRFP-SSTR5ΔCT in combination with increasing amounts of GFP-PIST were lysed and analyzed by Western blotting using anti-mRFP, or anti-PIST. Arrows point to the typical signal of the full-length receptor at 72 kDa and to an additional signal at lower molecular weight which was observed in PIST overexpressing cells. C. Quantitation of band intensities in B; overall levels of wt but not truncated receptor were increased in PIST overexpressing cells (*, p<0.05; ***, p<0.001; n = 3). Separate quantitation of both bands showed that the lower molecular weight band is most strongly increased by PIST expression. D. Lysates from cells expressing wt SSTR5 with GFP or GFP-PIST were treated with endo H or PNGase F deglycosylating enzymes, followed by Western Blot analysis. A typical of three experiments is shown.

As the PDZ ligand motif did not accelerate lysosomal targeting of the SSTR5, we reassessed the role of PIST, which had been implicated in lysosomal degradation of the CFTR. In agreement with previous work [8], PIST colocalizes with the SSTR5 at the Golgi apparatus; overexpressed PIST reduces the SSTR5-specific signal at the plasma membrane, and leads to formation of larger intracellular Golgi-like structures (Fig. 5A; see also Figure S3 in File S1). This effect is entirely dependent on the PDZ domain interaction, as the SSTR5ΔCT variant reaches the plasma membrane in the presence of overexpressed PIST. Upon analysis of the receptor in PIST overexpressing cells by Western blotting, we observed that PIST increased rather than decreased total cellular SSTR5 levels in a PDZ ligand dependent manner. PIST also leads to the appearance of a lower molecular weight form of the receptor, which accounts for most of the increase in total receptor signal (Fig. 5B,C). Deglycosylation experiments with PNGaseF and EndoH glycosidases showed that this lower molecular weight variant is sensitive to digestion with EndoH (Fig. 5D). Therefore we identify it here as an immature form of the receptor containing high mannose or hybrid carbohydrate modifications which are sensitive to EndoH [21]. This is consistent with a block of receptor maturation in an intermediate Golgi compartment upon PIST overexpression.

We asked whether PIST is involved in trafficking events from the TGN to the plasma membrane; in initial live cell imaging experiments we failed to observe dynamic movements of GFP-tagged PIST away from the Golgi apparatus, regardless whether the SSTR5 was coexpressed or not (data not shown). This was confirmed by imaging of cells expressing PIST tagged with the photoconvertible fluorescent protein Kaede. Here, conversion of green to red fluorescent PIST by a laser pulse directed at Golgi-localized PIST provided again no evidence for trafficking to the plasma membrane; instead, the red fluorescence only very gradually moved to other Golgi stacks within the same cell, without detectably moving to the cell surface (Figure S4 in File S1).

**Figure 6:**
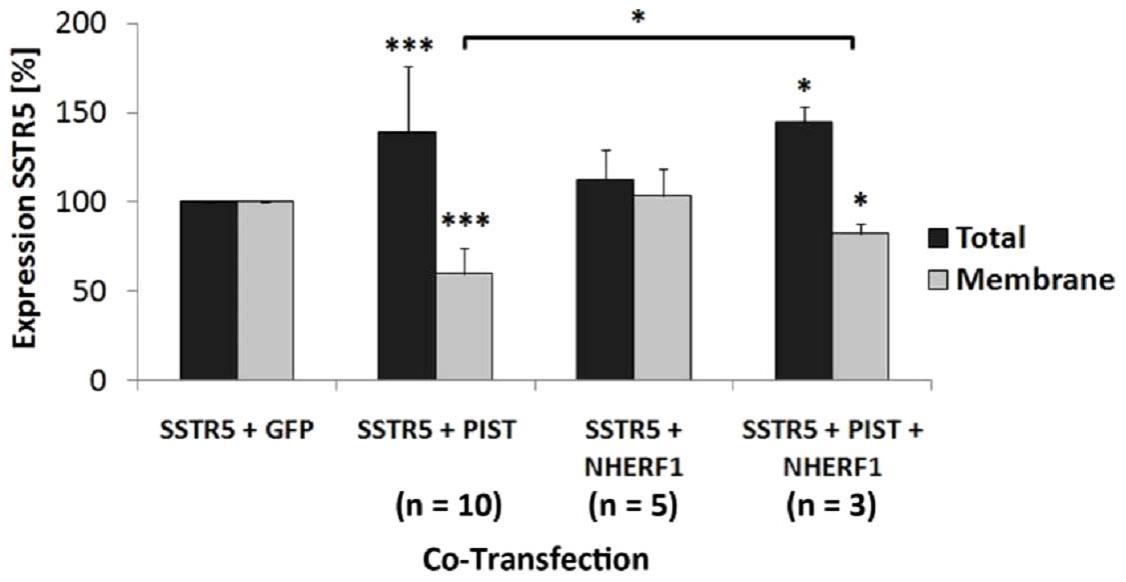
NHERF1 rescues cell surface availibility of SSTR5. Cells were transfected with vectors coding for mRFP-SSTR5 in combination with either GFP, GFP-PIST, or GFP-PIST in combination with GFP-NHERF1. After biotinylation of cell surface receptors, cells were lysed and biotinylated receptors were purified (ABC). Input and precipitate samples were analyzed by Western blotting using anti-RFP. The levels of surface SSTR5 (biotinylated receptors normalized on input) and total SSTR5 (normalized on GAPDH) is presented relative to the GFP expressing control condition. *,**,***, significantly different, p<0.05, 0.01, 0.001, respectively (n = 3).

The retention of SSTR5 in an intracellular compartment by PIST was quantitatively confirmed by cell surface biotinylation; PIST strongly increased the intracellular proportion of the receptor, i.e. the fraction of the receptor which is not accessible to cell surface biotinylation. As both NHERF1 and PIST interact with the PDZ ligand of the receptor, we asked whether NHERF1 can relieve the receptor from its intracellular anchorage by PIST. This was indeed the case, as coexpression of NHERF1 with PIST and the SSTR5 rescued the effect induced by PIST, in that the proportion of the receptor reaching the cell surface could be significantly increased (Fig. 6).

## Discussion

C-terminal motifs interacting with PDZ domains are found in many, but by far not all G-protein coupled receptors. This suggests that the PDZ ligand is not required for basal receptor functions, but may contribute additional functional features to any given receptor. This is exemplified here by the SSTR5, which does not require the PDZ motif for trafficking to the plasma membrane or agonist-dependent endocytosis. Internalization of GPCRs is triggered by agonist-induced binding of arrestins [3]; indeed β-arrestin-2 translocates to the plasma membrane after agonist treatment of SSTR5 expressing cells [22]. Here we show that the PDZ motif stabilizes SSTR5 by interfering with lysosomal degradation. This was evident upon analysis of biotinylated, endocytosed receptors where we observed that truncated receptors lacking the PDZ ligand motif are degraded faster than their wild type counterparts.

The SSTR5 PDZ ligand can bind to PDZ domains of PIST, SNX27 and NHERF1/3 proteins, all of which are expressed endogenously in HEK293 cells, with the exception of NHERF3/PDZ-K1. Importantly, these proteins are also coexpressed in several tissues and cell lines where SSTR5 is functional. This indicates that, similar to the situation in 293 cells, the receptor will be regulated by the interplay of all three types of PDZ proteins in native tissues. PIST has been described to induce targeting of the CFTR from the TGN to lysosomes, thereby reducing cellular CFTR levels [17], [18], [23]. As the affinity of the SSTR5 towards the PIST PDZ domain is rather high [19], we expected a similar or stronger effect for the SSTR5. Instead, our data show that PIST overexpression increases the overall cellular levels of SSTR5; taken together with the enhanced stability of endocytosed receptors carrying the PDZ motif, our data indicate that PIST is not involved in degradative processing of the SSTR5. This also suggests that lysosomal targeting of the CFTR by PIST requires additional sequence determinants (besides the PDZ ligand) in the CFTR which are not present in SSTR5.

SNX27 is associated with endosomes, and SSTR5 colocalizes with SNX27 in intracellular, vesicular structures likely to be endosomes. Knockdown of SNX27 by siRNA led to delayed postendocytic recycling of wt SSTR5, in agreement with several recent studies which demonstrated a role for SNX27 in the recycling of endocytosed β2-adrenergic receptors in HEK293 cells [4], [9]. SNX27 functions here as an essential adaptor for entry of receptors into retromer tubules which give rise to vesicular transport of receptors to the cell surface [4]. A more global analysis has shown that SNX27 prevents lysosomal entry of a large number of PDZ-ligand containing membrane proteins, and promotes their recycling instead [24]. However, it should be noted that in case of the SSTR5, the mutant receptor lacking the PDZ ligand can be efficiently recycled to the cell surface, in particular when lysosomal degradation is inhibited. This points to alternative recycling pathways for receptors with or without PDZ ligands. Indeed it has been described that additional sequence elements determine whether receptors use a non-selective, default pathway of recycling, which is independent of PDZ-mediated interactions [25]. More recently, it was shown that the recycling of PDZ ligand containing receptors may be negatively affected by protein interacting with C kinase 1 (PICK1), another PDZ protein which is attached to intracellular membranes [26]. Though we did not identify PICK1 as an interaction partner of the SSTR5 in our affinity purification approach (Figure S1 in File S1), it appears possible that PICK1 and SNX27 act antagonistically on receptor recycling.

In contrast to PIST and SNX27, NHERF1 and PDZ-K1 are scaffold proteins which interact with their target proteins at the plasma membrane [2]. NHERF proteins may integrate SSTR5 with other downstream signalling components such as G-protein subunits or potassium channels; recent work shows that PDZ-K1/NHERF3 enables coupling of the SSTR5 to phospholipase C-β3 [27]. Importantly, our data show that the function of NHERF1 is related to the role of PIST. PIST associates with the SSTR5 at the Golgi apparatus, presumably at the TGN [16], [28], [29]. In live imaging experiments in 293 cells expressing Kaede-PIST, we failed to observe movement of PIST away from the TGN. This is similar to our observations in cultured neurons [28]. Collectively our data indicate that PIST is stationary and may indeed retain receptors at the TGN, thereby preventing access to the cell surface. We observed an immature, EndoH sensitive glycosylation status of the SSTR5 in the presence of overexpressed PIST. As processing towards EndoH resistance occurs already in a medial Golgi compartment, this may indicate that PIST overexpression also inhibits progress of interacting membrane proteins through Golgi compartments. NHERF1 can release SSTR5 from retention at the TGN, and leads to a relative increase of cell surface accessible, functional SSTR5. This may be explained by binding of both PIST and NHERF1 to the PDZ ligand of SSTR5. A similar observation was made for cadherin-23, which binds to PIST at the TGN but is released to the plasma membrane by the PDZ proteins harmonin or MAGI-1 [29]. Interestingly, NHERF1 is present at rather low levels in mouse AtT20 cells, where we observed largely intracellular SSTR5 colocalizing with PIST [8], [30].

PIST binds to the PDZ ligands of more than 20 different membrane proteins, each of which has additional PDZ scaffolds which interact at the plasma membrane (e.g. SAP97 and MAGI in case of the β1-adrenergic receptor; NHERF family members in case of the CFTR [16], [31]. We hypothesize that PIST retains receptors at the Golgi until an appropriate PDZ scaffold is available to take the receptor to the plasma membrane. Thereby PIST would ensure that receptors are integrated into their signaling complexes at the membrane.

## Materials and Methods

### Expression constructs, cell lines and antibodies

mRFP-tagged mSSTR5 was expressed using a construct coding for a signal peptide (peptide sequence: MVLWLQLALLALLLPTSLAQGEVDI), followed by the mRFP cDNA (obtained from Roger Tsien, UCSD San Diego, CA; [32] and the mSSTR5 coding sequence as described [33]. A truncated construct lacking the four C-terminal amino acids (i.e. the PDZ ligand motif) was generated by PCR based methods. A cDNA coding for rat SNX27 was obtained from Paul Slesinger (Salk Institute, San Diego, CA) and subcloned into pEGFP-N1 (Clontech). The cDNA coding for human NHERF1 was obtained from Imagenes GmbH, Berlin, Germany, and subcloned into pEGFP-C3. Constructs coding for mouse PIST and PDZ-K1 as fusions with EGFP have been described [8].

For generation of stable, SSTR5-expressing cell lines, Flp-In 293 cells were obtained from Invitrogen (Leeks, The Netherlands). The SP-mRFP-SSTR5 expression cassette was subcloned into pEF5/FRT/V5-DEST vector; Flp-In cells were transfected with this vector, and clones of stable transfectants were selected in the presence of hygromycin B.

Antibodies were obtained from the following sources: mouse α-GFP: Covance; rat α-mRFP: Chromotek; mouse α-NHERF1: Enzo Life Sciences; mouse α-SNX27, Abcam. guinea-pig α-PIST has been described [8], [28].

### siRNA knockdown

Sets of four pre-designed siRNAs (FlexiTube; obtained from Qiagen, Hilden, Germany) were tested for silencing efficiency against SNX27 by transfection of HEK293 cells with 20 nmol siRNAs using RNAiMax (Invitrogen). Knockdown efficiency was determined by cell lysis and Western Blot 72 hours after transfection. siRNA #8 for SNX27 (Target sequence SNX27 #8: TACCAGATGGAACAACGGTTA) was most effective and used for further experiments. A non-targeting siRNA (AllStars Negative Control siRNA, Qiagen) was used as a negative control.

### Precipitation assays

Transfected cells were lyzed in RIPA buffer (50 mM Tris–HCl, pH 8.0, 150 mM NaCl, 1% NP-40, 0.5% Na-deoxycholate, 5 mM EDTA, 0.1% SDS, 0.2 mM phenylmethylsulfonyl fluoride, 1 µg/ml pepstatin, 10 µg/ml leupeptin) and centrifuged for 20 min at 20.000×g. Clear supernatants were incubated with either mRFP-trap or GFP-trap beads (Chromotek, Munich, Germany) for 2 hrs at 4°C. Beads were sedimented by centrifugation at 1000 g for 5 min, and washed four times with RIPA buffer. Aliquots of input and precipitate samples were analyzed by Western blot using appropriate antibodies. Chemiluminescent Western Blot signals were quantified by a ChemiDoc XRS luminescence imager (BioRad) in combination with Quantity One software. For isolation of proteins binding to the C-terminal PDZ ligand of SSTR5, the corresponding sequence was obtained as a synthetic peptide (NH_2_-ANGLMQTSRI-COOH) from EZBiolab Inc. (Westfield, IN, USA) and coupled covalently to NHS-sepharose (GE Healthcare) at a concentration of 3 mg/ml matrix. 50 µl of this matrix was used for affinity chromatography on HEK293 cell lysate in RIPA buffer, as described [34]. The C-terminal peptide of GKAP/SAPAP1 (NH_2_-IYIPEAQRTL-COOH) was used as negative control. Bound proteins were separated by SDS-PAGE, and analyzed by tryptic digestion, followed by mass spectroscopy [34].

### Live Cell Imaging

Transfected cells were plated on a 35 mm µ-Dish (Ibidi, Martinsried, Germany) and prior to microscopic analysis rinsed twice in HBSS and covered with prewarmed (37°C) imaging buffer (142 mM NaCl, 5.4 mM KCl, 1.8 mM CaCl_2_, 1 mM NaH_2_PO_4_, 25 mM HEPES, 5 mM glucose, 0.8 mM MgSO_4_, pH 7.4). Cells were visualized on a UltraVIEW VoX Spinning Disc microscope (Perkin Elmer, Rodgau, Germany) equipped with an incubation chamber (5% CO_2_, 37°C) using image analysis software Volocity 5 (Perkin Elmer).

### Cell surface biotinylation assays

Cell surface biotinylation assays were performed as described by [35]. Cells were washed three times in ice-cold HBSS and then exposed to 0.5 mg/ml Sulfo-NHS-SS-biotin (Pierce) for 30 minutes at 4°C. To quench excess Sulfo-NHS-SS-biotin, cells were washed three times in HBSS with 5 mM Tris (pH 7.4). Following this step, the procedure was adapted to the goal of the experiment. (1) For determination of cell surface receptors, cells were lyzed in RIPA buffer and centrifuged for 20 min at 20.000×g. Clear supernatants were incubated with EZview Red Streptavidin Affinity Gel (Sigma) for 4 hrs at 4°C. Beads were sedimented by centrifugation at 1000 g for 5 min, and washed four times with RIPA buffer. Aliquots of input and precipitate samples were analyzed by Western blot using appropriate antibodies. Chemiluminescent Western Blot signals were quantified by a ChemiDoc XRS luminescence imager (BioRad) in combination with Quantity One software. (2) for endocytosis: Cells were stimulated with 1 µM Somatostatin 28 (Bachem) in prewarmed (37°C) DMEM growth media for the appropriate time at 37°C. Remaining biotin label from cell surface proteins was removed by washing the cells twice in ice-cold glutathione stripping solution (50 mM glutathione, 75 mM NaCl, 1 mM EDTA, 10% FBS, 75 mM NaOH; 2× for 30 min at 4°C), followed by washing twice in ice cold HBSS. Cell lysis and quantitation of biotin-labeling was performed as described in 1.

(3) for degradation: Cells were stimulated with SST28 as above. Remaining biotin label from cell surface proteins was removed by washing the cells twice in ice-cold glutathione stripping solution (30 min at 4°C). Glutathione-stripped cells were again incubated with prewarmed growth media for 3 h or 6 h in a CO_2_ incubator at 37°C, followed by cell lysis and quantitation of biotin-labeling.

(4) for recycling: Cells were stimulated with SST28 for 15 min as above, followed by stripping with glutathione. Cells were rewarmed to 37°C in DMEM with 10% FBS for varying lengths of time in a CO_2_ incubator to permit recycling. A second glutathione treatment as described before was performed to remove any biotin from biotinylated receptors that have recycled back to the plasma membrane during the second 37°C incubation. Cell lysis and quantitation of biotin-labeling was performed as described in 1.

## Supporting Information

File S1 **Figures S1–S4 in Supporting Information File S1. Figure S1. Purification of SSTR5 interacting proteins.** 293 cells were lysed in RIPA buffer; cleared lysates were subjected to affinity chromatography on control peptide conjugated beads (GKAP C-terminal PDZ ligand; left lane) or SSTR5 C-terminal peptide (right lane). Purified proteins were separated by SDS gel electrophoresis, followed by staining with Coomassie Brilliant Blue. Bands that were specific to the SSTR5 sample (see arrows) were cut out, digested by trypsin, and analyzed by mass spectrometry. Identity of these bands is shown in the table. Note that sorting nexin 27 constitutes one of the strongest bands; MPDZ (also known as MUPP1) was not considered further because the band was very weak. **Figure S2. Expression pattern of SSTR5 interacting proteins.** Cell lines and tissues relevant to SSTR5 function were prepared and lyzed in RIPA buffer; equal amounts were analyzed by Western blotting using the antibodies indicated. **Figure S3. HEK293T cells were cotransfected with SP-RFP-SSTR5 and SNX27-GFP (A) or PIST-GFP (B).** The localization of the proteins in the cell was detected in live cell imaging experiments using a *Perkin-Elmer Spinning Disc* Microscope. Note the high amount of colocalization of SNX27 with the receptor in A, and the almost perfect colocalization with PIST in B. Scale bar: 10 µm. **Figure S4. Live cell imaging of Kaede-PIST.** 293 cells expressing Kaede-tagged PIST were mounted on a live imaging stage (37°C; 5% CO_2_) of a spinning disc confocal microscope. A single cell was irradiated in the area indicated by a circle (time 0:00 minutes) by a short (25 cycles of 20 msec each) 405 nm laser pulse, thereby converting green Kaede fluorescence to red. The cell was then imaged for 14 minutes, using settings for green (A,C,E,G,I,K,M,O) and red (B,D,F,H,J,L,N,P) fluorescence. Note that most of the red fluorescent signal stays in the irradiated area during the observation period, whereas only some signal moves out to the other Golgi-like structures which are labeled by green fluorescence.
(DOCX)
