# Supplementary material for: Subcellular Sorting of the G-Protein Coupled Mouse Somatostatin Receptor 5 by a Network of PDZ-Domain Containing Proteins
Source: PLoS One. 2014 Feb 11;9(2):e88529. doi: 10.1371/journal.pone.0088529 (PMC3921201; doi:10.1371/journal.pone.0088529)
Supplement: File S1 — Figures S1–S4 in Supporting Information File S1. Figure S1. Purification of SSTR5 interacting proteins. 293 cells were lysed in RIPA buffer; cleared lysates were subjected to affinity chromatography on control peptide conjugated beads (GKAP C-terminal PDZ ligand; left lane) or SSTR5 C-terminal peptide (right lane). Purified proteins were separated by SDS gel electrophoresis, followed by staining with Coomassie Brilliant Blue. Bands that were specific to the SSTR5 sample (see arrows) were cut out, digested by trypsin, and analyzed by mass spectrometry. Identity of these bands is shown in the table. Note that sorting nexin 27 constitutes one of the strongest bands; MPDZ (also known as MUPP1) was not considered further because the band was very weak. Figure S2. Expression pattern of SSTR5 interacting proteins. Cell lines and tissues relevant to SSTR5 function were prepared and lyzed in RIPA buffer; equal amounts were analyzed by Western blotting using the antibodies indicated. Figure S3. HEK293T cells were cotransfected with SP-RFP-SSTR5 and SNX27-GFP (A) or PIST-GFP (B). The localization of the proteins in the cell was detected in live cell imaging experiments using a Perkin-Elmer Spinning Disc Microscope. Note the high amount of colocalization of SNX27 with the receptor in A, and the almost perfect colocalization with PIST in B. Scale bar: 10 µm. Figure S4. Live cell imaging of Kaede-PIST. 293 cells expressing Kaede-tagged PIST were mounted on a live imaging stage (37°C; 5% CO2) of a spinning disc confocal microscope. A single cell was irradiated in the area indicated by a circle (time 0:00 minutes) by a short (25 cycles of 20 msec each) 405 nm laser pulse, thereby converting green Kaede fluorescence to red. The cell was then imaged for 14 minutes, using settings for green (A,C,E,G,I,K,M,O) and red (B,D,F,H,J,L,N,P) fluorescence. Note that most of the red fluorescent signal stays in the irradiated area during the observation period, whereas only some signal moves out [file pone.0088529.s001.docx]

**Figure S1**

| **Protein** | **Short name** | **Accession number** | **Mass (kDa)** | **PDZ domains** |
| --- | --- | --- | --- | --- |
| PDZ protein interacting specifically with TC10 | PIST | Q9HD26 | 51 | 1 |
| Sorting nexin-27 | SNX27 | Q96L92 | 61 | 1 |
| Amyloid protein-binding protein 2 | APBP2 | Q92624 | 67 | 0 |
| Heat shock 70 kDa protein 1A/1B | HSP71 | P08107 | 70 | 0 |
| 78 kDa glucose-regulated protein | GRP-78 | P11021 | 72 | 0 |
| Stress-70 protein, mitochondrial | GRP-75 | P38646 | 74 | 0 |
| Multiple PDZ domain protein | MPDZ | O75970 | 222 | 13 |

**Figure S1**. **Purification of SSTR5 interacting proteins.** 293 cells were lysed in RIPA buffer; cleared lysates were subjected to affinity chromatography on control peptide conjugated beads (GKAP C-terminal PDZ ligand; left lane) or SSTR5 C-terminal peptide (right lane). Purified proteins were separated by SDS gel electrophoresis, followed by staining with Coomassie Brilliant Blue. Bands that were specific to the SSTR5 sample (see arrows) were cut out, digested by trypsin, and analyzed by mass spectrometry. Identity of these bands is shown in the table. Note that sorting nexin 27 constitutes one of the strongest bands; MPDZ (also known as MUPP1) was not considered further because the band was very weak.

**Figure S2**

**
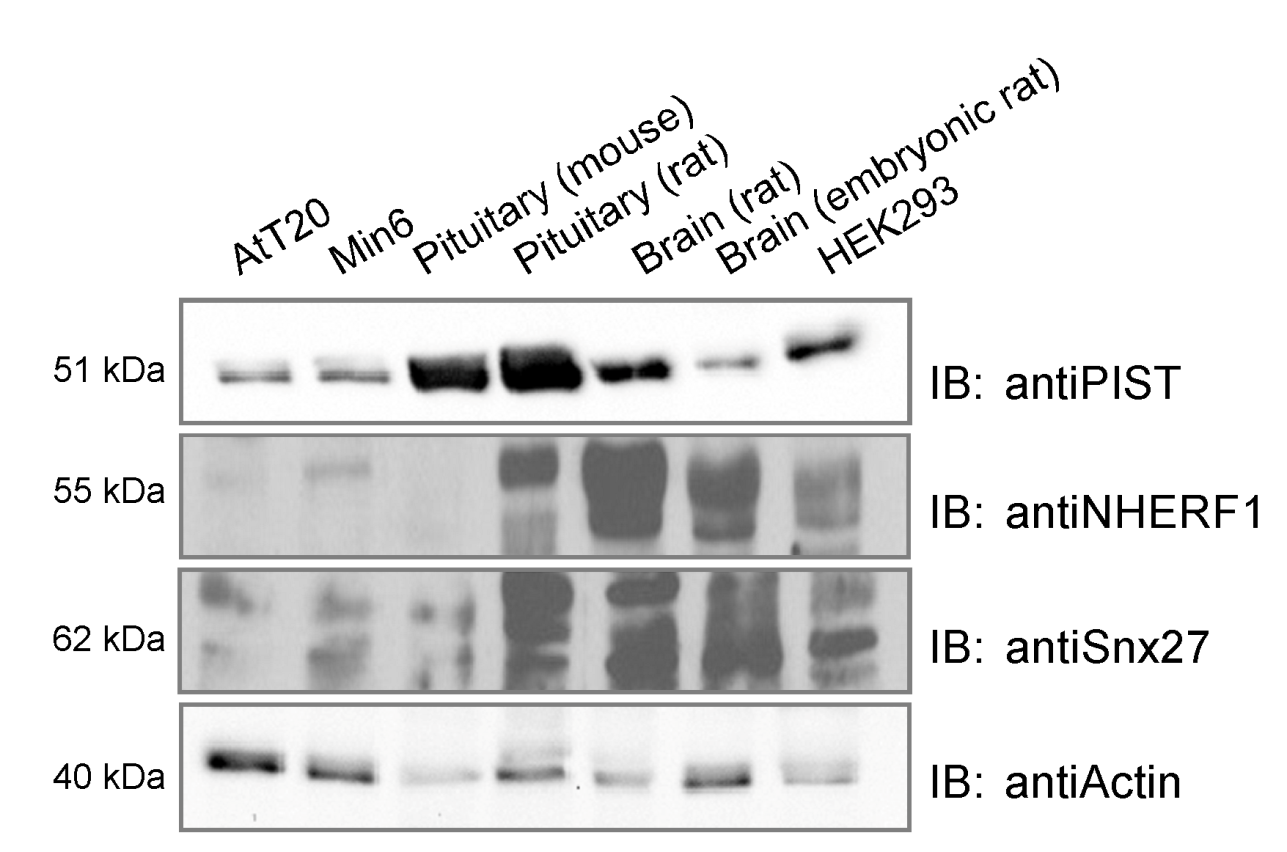
**

**Figure S2. Expression pattern of SSTR5 interacting proteins.** Cell lines and tissues relevant to SSTR5 function were prepared and lyzed in RIPA buffer; equal amounts were analyzed by Western blotting using the antibodies indicated.

**Figure S3**

A
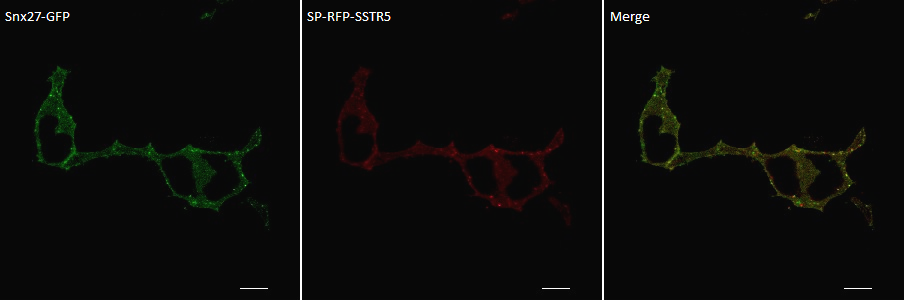


B


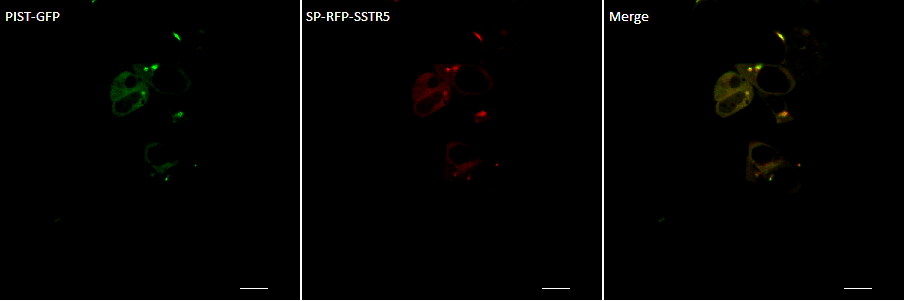


**Figure S3**. HEK293T cells were cotransfected with SP-RFP-SSTR5 and SNX27-GFP (A) or PIST-GFP (B). The localization of the proteins in the cell was detected in live cell imaging experiments using a *Perkin-Elmer Spinning Disc* Microscope. Note the high amount of colocalization of SNX27 with the receptor in A, and the almost perfect colocalization with PIST in B. Scale bar: 10µm

**Figure S4**

**
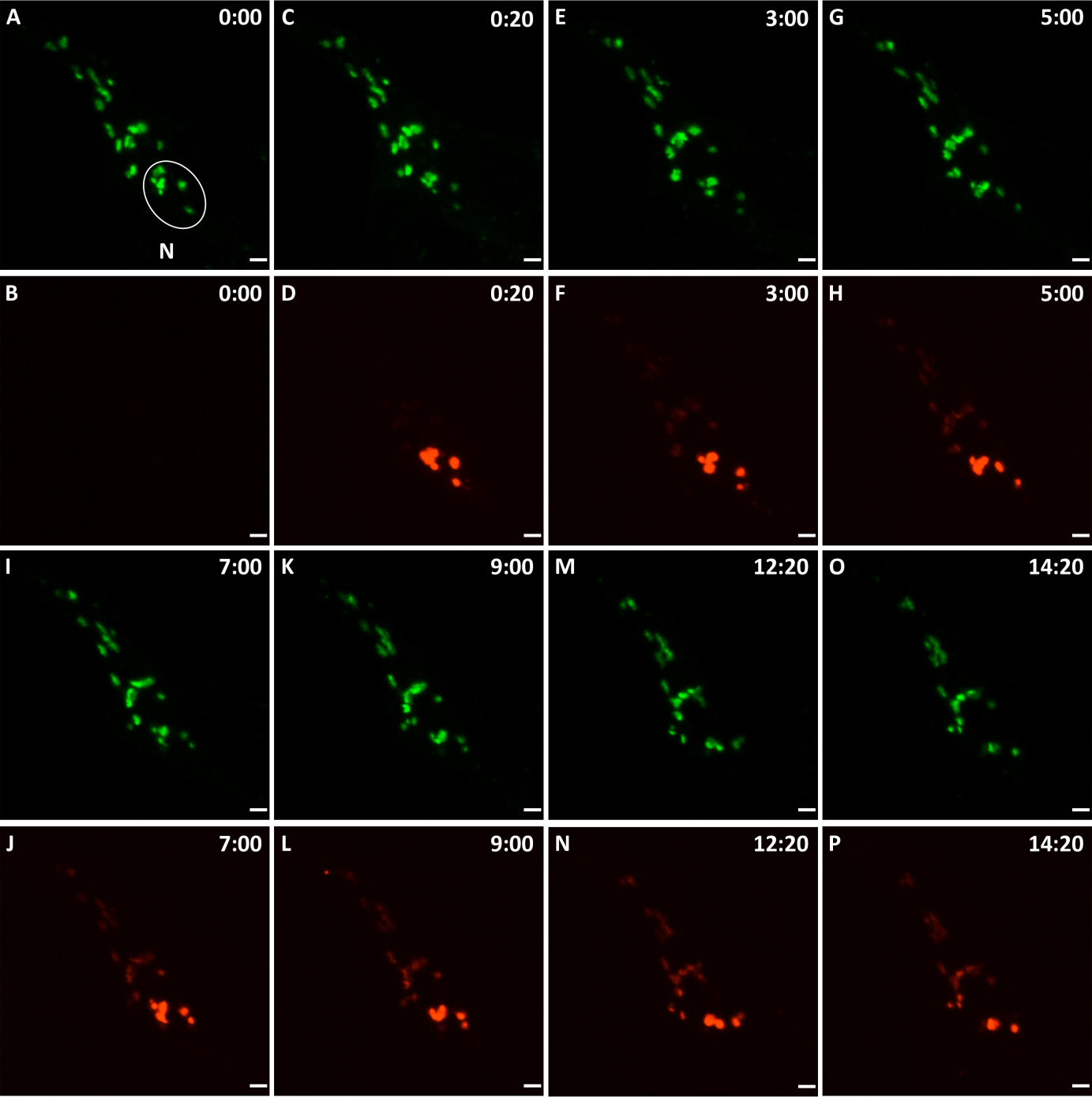
**

**Figure S4**. **Live cell imaging of Kaede-PIST.** 293 cells expressing Kaede-tagged PIST were mounted on a live imaging stage (37°C; 5 % CO_2_) of a spinning disc confocal microscope. A single cell was irradiated in the area indicated by a circle (time 0:00 minutes) by a short (25 cycles of 20 msec each) 405 nm laser pulse, thereby converting green Kaede fluorescence to red. The cell was then imaged for 14 minutes, using settings for green (A,C,E,G,I,K,M,O) and red (B,D,F,H,J,L,N,P) fluorescence. Note that most of the red fluorescent signal stays in the irradiated area during the observation period, whereas only some signal moves out to the other Golgi-like structures which are labeled by green fluorescence.
